# Application status and optimization suggestions of tumor organoids and CAR-T cell co-culture models

**DOI:** 10.1186/s12935-024-03272-x

**Published:** 2024-03-05

**Authors:** Rong-Xuan Ning, Cun-Yu Liu, Shi-Qi Wang, Wen-Kai Li, Xia Kong, Zhi-Wei He

**Affiliations:** 1 The First Dongguan Affiliated Hospital, Guangdong Medical University, No. 42 Jiaoping Road, Tangxia Town, Dongguan, 523710 Guangdong Province China; 2 China-America Cancer Research Institute, Guangdong Medical University, Dongguan, 523808 Guangdong Province China; 3 School of Basic Medicine, Guangdong Medical University, Dongguan, 523808 Guangdong Province China

**Keywords:** Tumor organoid, CAR-T therapy, Co-culture models

## Abstract

Tumor organoids, especially patient-derived organoids (PDOs) exhibit marked similarities in histopathological morphology, genomic alterations, and specific marker expression profiles to those of primary tumour tissues. They are applied in various fields including drug screening, gene editing, and identification of oncogenes. However, CAR-T therapy in the treatment of solid tumours is still at an exploratory stage. Tumour organoids offer unique advantages over other preclinical models commonly used for CAR-T therapy research, which the preservation of the biological characteristics of primary tumour tissue is critical for the study of early-stage solid tumour CAR-T therapies. Although some investigators have used this co-culture model to validate newly targeted CAR-T cells, optimise existing CAR-T cells and explore combination therapy strategies, there is still untapped potential in the co-culture models used today. This review introduces the current status of the application of tumour organoid and CAR-T cell co-culture models in recent years and commented on the limitations of the current co-cultivation model. Meanwhile, we compared the tumour organoid model with two pre-clinical models commonly used in CAR-T therapy research. Eventually, combined with the new progress of organoid technologies, optimization suggestions were proposed for the co-culture model from five perspectives: preserving or reconstructing the tumor microenvironment, systematization, vascularization, standardized culture procedures, and expanding the tumor organoids resource library, aimed at assisting related researchers to better utilize co-culture models.

## Introduction

Since the pioneering work of Sato et al. [1] in constructing colorectal cancer organoid models, there has been significant progress in the development of tumor organoids. The establishment and advancement of tumor organoids have provided a novel approach for creating more physiologically relevant human tumor models. In comparison to commonly used cancer models like cancer cell lines and primary patient-derived tumor xenografts (PDTXs), tumor organoids offer distinct advantages. The advantages have resulted in their utilization across diverse domains, encompassing drug screening, genome editing, and oncogene identification [2].

Chimeric Antigen Receptor T-Cell Immunotherapy (CAR-T therapy) has garnered significant interest as a burgeoning treatment for tumors, demonstrating promising outcomes in the management of specific hematological tumors such as B-cell acute lymphoblastic leukemia (B-ALL), B-cell non-Hodgkin lymphoma (B-NHL), and multiple myeloma (MM) [3–5]. The scientific community has shown considerable attention towards the efficacy of CAR-T therapy in addressing hematological tumors, perceiving it as a potential remedy for solid tumors. Nevertheless, the effectiveness of this therapy is constrained by factors such as tumor heterogeneity, limited transport and infiltration of CAR-T cells into tumor tissues, and the presence of immunosuppressive microenvironments within the tumor [6]. These crucial concerns emphasise the necessity of preclinical research models for CAR-T therapy.

Tumor organoids can preserve primary tumour tissue characteristics more completely, allowing for a realistic simulation of the interaction between tumour and CAR-T cells in vitro. Researchers have co-cultured CAR-T cells with tumor organoids to validate the anti-tumour effects of new target CAR-T cells, modified CAR-T cells, and CAR-T combination therapies.

However, there remains a significant amount of unexplored research potential within the currently utilized co-culture models of tumor organoids and CAR-T cells. This review summarizes a total of 10 research papers on the use of tumor organoids for CAR-T therapy from March 2019 to June 2023. At the same time, based on the new advances in organoid technologies from September 2014 to June 2023, optimization suggestions were proposed for this co-culture model from five perspectives: preserving or reconstructing the immune microenvironment, systematization, vascularization, standardized culture procedures, and expanding the tumor organoid resource library. All cited articles are sourced from the Pubmed database. We hope that this review will offer a fresh perspective to assist CAR-T therapeutics researchers in the successful implementation of this co-culture model in both fundamental and translational CAR-T therapies.

## A basic model for co-culture of tumor organoids with CAR-T cells

As of June 2020, there are currently over 500 ongoing clinical trials investigating CAR-T therapy for various tumor types, with numerous additional trials in the developmental phase. The development of a practical, cost-effective, and realistic in vitro model for early CAR-T research is of utmost importance as it allows for the evaluation of the anti-tumor activity of CAR-T cells, a detailed exploration of the mechanism of action of CAR-T cells, and an enhancement of the structural design of CAR-T cells. Furthermore, there is a growing clinical demand for personalized medicine models that can predict the efficacy of CAR-T treatments and identify viable combinations of CAR-T therapy strategies. Co-culture models of tumor organoids have become a prominent choice owing to their remarkable resemblance to the original tumor in terms of histopathological morphology, genomic alterations, and expression profiles of specific markers.

**Fig. 1:**
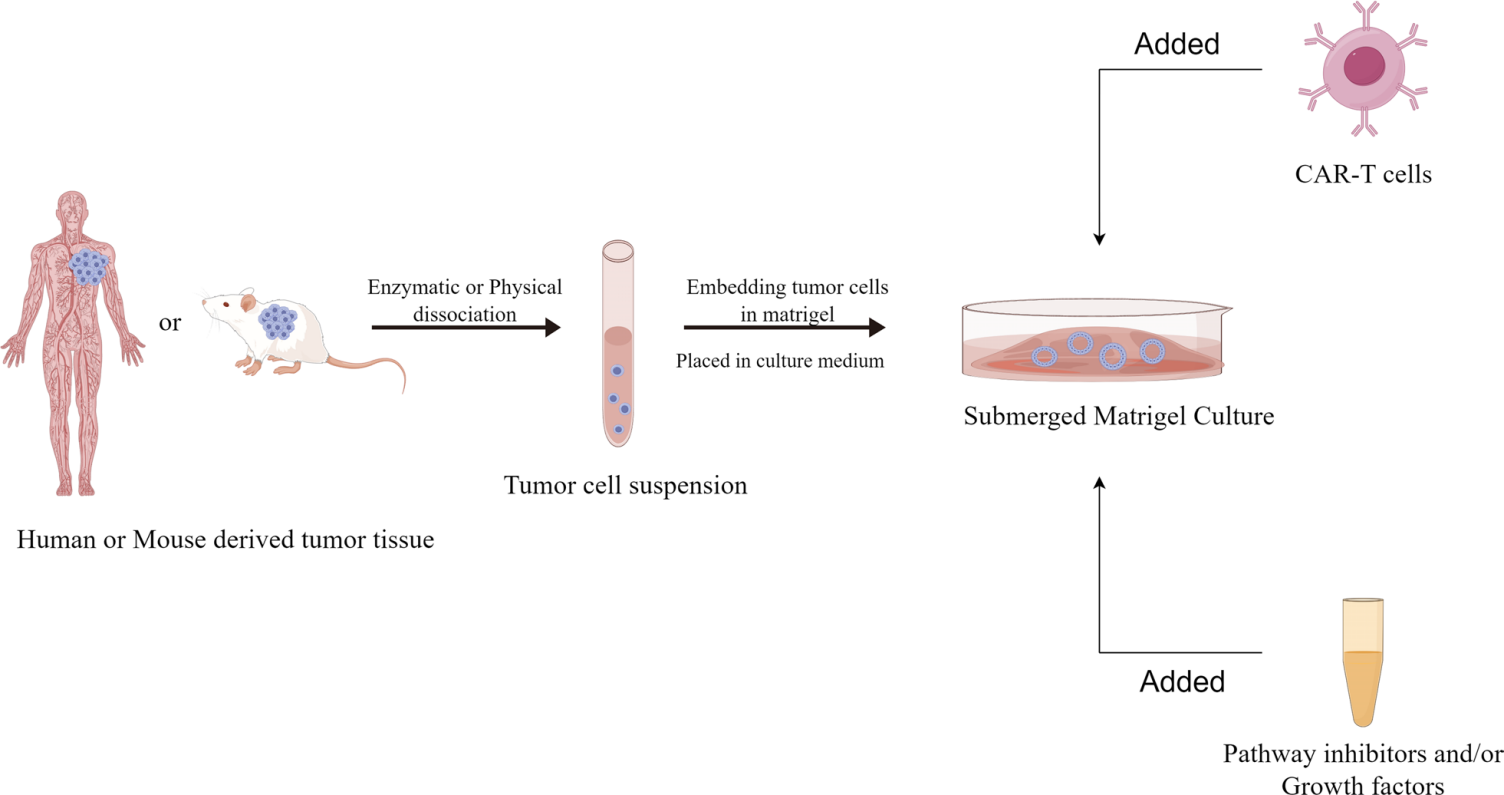
Preparation process of tumor organoids and CAR-T co-culture basic model (by Figdraw.)

Submerged Matrigel culture is a classic approach for organoid culture and is applicable to tumor organoids [7]. This method involves dissociating tumor tissue into a dispersed suspension of tumor cells using enzymatic or physical techniques. These cells are then embedded in a gel and placed in a culture medium to facilitate the growth of tumor organoids. The culture medium not only contains essential components for organoid growth but also includes pathway inhibitors and/or growth factors. And the additives and culture conditions being adapted based on the tumor organoids type [8–10]. Common additions comprise Wnt3a, R-spondin, epidermal growth factor (EGF), and Noggin—a bone morphogenesis (BMP) inhibitor which together promote stem cell growth, differentiation, and self-renewal [11] (Fig. 1)

It should be emphasised that the application of the Submerged Matrigel culture approach for tumor organoids is not fixed and should be customized to suit the unique attributes of the tumor organoids. Jacob et al. [12] generated glioblastomas organoids by directly culturing micro-dissected tumor fragments, as opposed to dispersing the tumor tissues into cell suspensions. Tumour fragments were manipulated directly to ensure the survival of sensitive cells and reproduced the hypoxic gradient, whilst preserving part of the mesenchymal component in tumor organoids. This facilitated the study of tumour cell-mesenchymal cell interactions. Fujiii et al. [10] discovered that genetic mutations in tumour cells had a tendency to impact the nature of the additive. The majority of colorectal cancer (CRC) organoids and all adenoma organoids were capable of autonomous growth without the need for exogenous R-spondin/Wnt3a. This can be attributed to the existence of mutations in one of the pivotal protein genes involved in the Wnt signalling pathway, which sustains the activation of said pathway. For example, variation-induced activation of TCF7L2, CTNNB1, APC. Hence, it is imperative to establish the requirement for growth factor/pathway inhibitors based on the genetic characteristics of the tumor organoids. Incorrect application of growth factor/pathway grafts may result in induced clonal selection of the tumor organoids, while the incorporation of incorrect components may complicate the interpretation of drug therapy [12]. The instructions/literature should be read in detail to determine the appropriate culture procedure and additives for the use of Submerged Matrigel culture for tumor organoids.

How can the anti-tumour activity of CAR-T cells be accurately analyzed in a co-culture model? Three studies offer valuable insights into this inquiry. Zou et al. [13] utilised a Caspase3/7 green fluorescent probe to label tumor organoid cells undergoing apoptosis, and also employed flow cytometry to quantitatively analyse CAR-T cells with elevated killing virulence expressing CD107a, IFN-γ. Schnalzger et al. [14] performed lentiviral transduction of luciferase/GFP into tumor organoids. They quantified the anti-tumour activity of CAR-T cells based on the remaining fluorescence intensity, which decreased as tumor organoid cells died. Additionally, Yu et al. [15] labelled CAR-T cells with CD8 and granzyme B, observing differences between the two markers in experimental and control groups. LDH, IL-2, TNF-α, and IFN-γ in the cell matrix were quantified. In brief, the evaluation of the anti-tumour activity of CAR-T cells comprises three perspectives: apoptosis of tumor organoids, killing activity of CAR-T cells, and the content of relevant cytokines in the cell matrix.

## Current application of tumor organoids and CAR-T cell co-culture models

### Validation of the anti-tumour effect of newly targeted CAR-T cells

CAR-T therapy has demonstrated promising outcomes in specific haematological malignancies, but its efficacy in solid tumours is considerably limited. Despite the identification of several potential targets, it is crucial to investigate novel targets in order to advance the field [16]. Therefore, there is an urgent need for a preclinical model that can faithfully replicate the unique surface markers found on human cells and can be readily constructed to assess the therapeutic efficacy of CAR-T on new targets. In this regard, tumor organoids offer a viable solution.

Yu [15] and Jacob [12] independently developed organoid models for bladder cancer and glioblastoma, and proved the degree of preservation of tumor organoids on biological characteristics of primary tumor tissue. Subsequently, they conducted co-culturing experiments by introducing MUC1-CAR-T cells and EGFR VIII-CAR-T cells to their respective tumor organoids, thereby verified the anti-tumorigenic effect of these two innovative CAR-T cell targets. Tumors overexpressing MET are generally insensitive to small molecule targeted drugs therapy. In light of this, Chiriaco et al. [17] devised two MET-CAR constructs with distinct structures specifically for MET-overexpressing tumours, and assess the anti-tumour effect of these two constructs by utilising different tumour organoid models that overexpress MET. Both types of MET-CAR-T cells can overcome resistance to small molecule targeted drugs against MET, and their anti-tumor activity is correlated with MET expression levels. Li et al. [18] co-cultured NSCLC organoids expressing B7-H3 with B7-H3-CAR-T cells to verify their anti-tumor activity prior to brain metastasis. Then, CCR2b was expressed on the CAR-T cell surface. Finally, it was confirmed in the patient-derived tumor xenograft (PDTX) model that its binding with CCR2 on the surface of tumor cells can promote CCR2b-B7-H3-CAR-T cells to penetrate the blood–brain barrier.

### Optimise existing CAR-T cells

The identification of novel targets broadens the potential applicability of CAR-T therapy, and on this basis, it is also meaningful to optimise existing CAR-T cells in multiple ways to enhance their killing effect. The presence of tumor organoids also provides a platform for evaluating the anti-tumour effect of CAR-T after optimisation.

The optimization of CAR-T cells can be undertaken from various perspectives, often involving the modification of the CAR structure to enhance the cytotoxicity of CAR-T cells. Thokala et al. [19] replaced the single stranded fragment variable region (scFv) of CD19-CAR-T cells with monoclonal antibody (mAb) 806, which can target various EGFR mutants. In order to evaluate the anti-tumor efficacy of improved CAR-T cells, researchers co-cultured them with GBM organoid containing multiple EGFR mutations. The improved CAR-T cells successfully targeted and eliminated multiple tumor copies, significantly reducing the possibility of escaping tumors through antigen loss. Wang et al. [20] constructed a modified CAR-T cell targeting glypican-3 (GPC3) in hepatocellular carcinoma (HCC).This modification involved substituting the cd8α-derived hinge region in the conventional CAR structure with a 4-1bb-derived hinge region containing 11 cysteine residues. By co-culturing with HCC organoids, it was observed that modified CAR-T cells showed stronger effectiveness in inhibiting tumor growth.

Zou et al. [13] attempted to optimize CAR-T therapy by screening a subset of CAR-T cells with strong anti-tumor activity. Researchers divided HBVs CAR-T cells into two groups based on whether express CD39. Subsequently, co-culturing these populations with HBV + HCC organoid models revealed that CD39 + HBVs CAR-T cells exhibited more significant apoptosis induction in HCC organoids. Therefore, CD39 can serve as an indicator to distinguish the subgroups of CAR-T cells with stronger activity in HBV + HCC.

Qiao et al. [21] restored the killing ability of CAR-T cells by disabling immune checkpoints and immunosuppressive molecule receptors. Specifically, they developed two CAR-T cells targeting EGFR and B7-H3 in cholangiocarcinoma tissue. Subsequently, shRNA was used to knockout programmed cell death protein 1 (PD-1), T cell immunoglobulin and mucin domain protein 3 (Tim-3), T lymphocyte immunoglobulin, ITIM domain (Tigit), and transforming growth factor β Receptors (TGF-β r), interleukin-10 receptor (IL-10R) and interleukin-6 receptor (IL-6R). The efficacy of these CAR-T cells in combating cholangiocarcinoma organoids was evaluated through co-culture experiments. Knockout CAR-T cells exhibit stronger anti-tumor activity. This discovery provides a new approach to reactivate the cytotoxicity of CAR-T cells against solid tumors while reducing CAR-T cell depletion.

### Exploring combination therapy strategies

In GBM, the failure of CAR-T therapy can be attributed to the phenomenon of antigenic escape of EGFRVIII targets. Thokala et al. [19] proposed a solution to this problem by making modifications to the structure of CAR-T cells. Conversely, Song et al. [22] directed their attention towards the IAP inhibitor birinapant. Birinapant can activate NF-κB pathway enhances the killing ability of CAR-T cells and increasing the sensitivity of tumour cells to cytokines secreted by CAR-T cells, ultimately leading to apoptosis. Introducing birinapant into the co-culture model of GBM organoid and EGFR VIII CAR-T cells demonstrated a strong ability to clear tumors. This method effectively limits the occurrence of antigen escape in GBM. This strategy of combining small molecule drugs with CAR-T therapy provides new ideas for the immunotherapy of solid tumors.

### Limitations of the tumor organoids and CAR-T co culture model currently used in research

Tumor organoids have certain advantages over traditional CAR-T preclinical models, so they have been used by some researchers as a supplement to traditional CAR-T preclinical models. However, the above literature on this co-culture model still has limitations. It is important to fully understand these limitations and make improvements to better serve future research on solid tumor CAR-T therapy.

The final quality of tumor organoids can be affected by various stages of the construction process. The tumor organoids we construct need to faithfully reflect the biological characteristics of the primary tumor tissue, which is also one of the important principles of tumor organoids construction. Only some articles evaluate the preservation of tumor organoids by comparing changes in their genetic material. This operation provides a guarantee for the subsequent validation of anti-tumor effects by co-culturing tumor organoids with CAR-T cells.

Secondly, and most importantly. The killing ability of CAR-T cells, whether in clinical or preclinical studies, largely depends on the tumor microenvironment (TME).Therefore, the evaluation of the anti-tumor effect of CAR-T cells in co-culture models cannot exclude TME as an important factor. The tumor organoid culture models used in the above articles are basic models and cannot fully preserve the TME of primary tumor tissue. This mean that the evaluation of the anti-tumor effect of CAR-T cells is conducted without the presence of TME, which cannot accurately represented anti-tumor effect of CAR-T cells.

Finally, the vascular tissue of the tumour is also a key factor affecting the infiltration effect of CAR-T cells. It is crucial to realistically simulate the physiological process of CAR-T cells migrating from blood vessels and acting on local tumour tissues, especially for larger tumours organoids. The above article only embeds CAR-T cells and tumor organoids in a matrix gel, attracting CAR-T cells to infiltrate the local area through tumor organoids without the key link of CAR-T cell migration from blood vessels. This ultimately leads to differences between in vitro and in vivo experimental results.

## Comparison of tumor organoids with other preclinical models in CAR-T therapy research

Frequently employed tumor models in studies on CAR-T therapy encompass cancer cell lines and primary patient-derived tumor xenograft models (PDTXs).Tumor organoids complement the above two models in some places, so some researchers have utilized tumor organoids in the early stages of CAR-T treatment research. In Table 1, we will compare this co-culture model with commonly used preclinical models in CAR-T therapy research.

**Table 1:** Comparison of preclinical models commonly used in CAR-T therapy reaserch

	Cancer cell lines	Tumor organoids	PDTXs
Richness of variety	Various	Medium	Low
Heterogeneity	+	+++	++
Difficultly of culture	Easy	Medium	Difficult
Resource consumption	Low	Low	High
High-throughput drugs screening	++	+++	−
Similarity with primary tumors	+	+++	++
Success rate of construction	+	+++	++
Gene Editing	++	++	+ Editing on embryonic stem cells
Presence of TME	−	++ Can be preserved or reconstruction	++

Respective features were judged as Very suitable (+++), Overall suitable (++), Possible suitability (+), unsuitable (−). PDTXs (patient-derived tumour xenograft)

The table above shows that each of the three tumor models has its own advantages and complements the others. In CAR-T therapy research, it is not appropriate to choose a single tumor model for efficacy validation. The selection should be based on the principle of from simple to complex, in vivo and in vitro experiments complement each other. Specifically, although tumor cell lines have limited complexity, lack heterogeneity, and cannot reflect the side effects of CAR-T therapy, they have advantages in culture, rapid growth, and good economic viability. Therefore, if tumor cell lines express target molecules of CAR-T cells, it can be used for early validation of the anti-tumor effect of CAR-T cells. Tumor organoids and PDTXs have significant advantages in heterogeneity, preservation of original tumor features, and preservation of TME. Therefore, it is possible to add the above two models for validation based on the good results of cancer cell line validation.

## Optimization suggestions for the co-culture model of tumor organoids and CAR-T cells

### Preservation or reconstitution of the tumour microenvironment (TME) in co-culture models

The Tumour Microenvironment (TME) comprises a multifaceted cellular milieu surrounding the tumour epithelium. It encompasses diverse constituents such as fibroblasts, blood vessels, infiltrating immune cells, and extracellular matrix, among others [23]. The constrained effectiveness of CAR-T therapies against solid tumours is intimately linked to the TME. The impact of the TME manifests in limited transport of CAR-T cells to the tumour, inadequate infiltration into the tumour, shortened lifespan, and exposure to various immunosuppressive signals [6]. The true efficacy of CAR-T cells in eradicating tumors cannot be adequately demonstrated using tumor organoids that lack the TME. The integration of TME into tumor organoids models establishes a foundation for investigating the interaction between CAR-T cells and TME. However, the deficiency of TME is a limitation in the aforementioned researches.

However, the question of how to introduce the TME in organoid models is a crucial one, and a multitude of scholars have proffered their respective resolutions. Dijkstra et al. [24] conducted a study wherein they utilized mismatch repair gene-deficient colorectal cancer (dmmR CRC) organoids and NSCLC organoids, which were co-cultivated with PBMC. Following a two-week culture period, the researchers observed a notable increase in the CD8+ T-cell population. This study establishes a foundation for reconstituting tumour organoid TMEs by adding immune components to Submerged Matrigel Culture. The deliberate integration of one or more components into PDOs serves to mitigate the influence of external components on the analyzed component. Therefore, this model displays potential for scientific investigation into the mechanism of action of CAR-T cells in the TME. Nonetheless, the construction of the model solely through the addition of exogenous immune components falls short of fully simulating the authentic TME. Neal et al. [25] developed the Air Liquid Interface Culture method, where tumour cells and mesenchymal stromal cells were cultured together without reconstruct. Air Liquid Interface Culture method’s ability to preserve TME is dependent on the direct contact between the surface of the matrix gel and air. This improves the gas exchange of tumor organoids in the matrix gel and promotes the formation of oxygen concentration gradients. This not only facilitates the growth of tumor organoids towards larger volumes, but also maintains immune cell activity and function. They confirmed that TILs in the model retained the original TCR expression profile of the tumour cells and emulated immune checkpoint blockade through the activation of Tumour-specific TILs using anti-PD-1/PD-L1.In order to better simulate the local environment of tumour tissues in in vitro organs in vivo, Sontheimer-Phelps et al. [26] have developed the Microfluidic 3D culture method for organoid culture, this technique not only maintains the tumor microenvironment (TME), but also reconstructs the intricate three-dimensional tissue structure of the organ through the utilization of microfluidics. By simulating mechanical forces such as tissue deformation, hydrostatic pressure, and fluidic shear stress, this method successfully emulates the complex physiological conditions experienced by the organ. The Microfluidic 3D culture method more accurately replicates the pathological conditions of tumor tissues in vivo, while simultaneously preserving the typical mechanical activities observed within the organs. At the cellular level, Microfluidic 3D culture effectively preserves in vivo cell–cell and cell–matrix interactions. Specifically, it simulates the interactions between immune cells-tumor cells and immune cells-immune cells in TME, while providing appropriate extracellular matrix structure and mechanical properties for tumor cells and immune cells. This greatly supports the infiltration of immunological cells in tumor tissue.

In general, there are developed techniques for reconstructing or preservation TME in organoids, reconstructing or preserving the co-culture model of TME will become an inevitable trend in future CAR-T therapy research.

### Systematising co-culture models for toxicity assessment of CAR-T therapies

Oncology patients treated with CAR-T therapy for solid tumours will inevitably suffer from toxicities [27, 28]. Of these, no-target, off-tumour toxicity (OTOT) is of utmost clinical concern [29]. However, in vitro models are not the preferred approach for evaluating the toxicity of CAR-T therapy due to the absence of normal cells and a lack of systematization. Although the in vivo model represented by mice overcomes the above drawbacks, the significance of using the in vivo model for the assessment of toxicities is also very limited, including the structure and expression of the tumour-associated antigen (TAA), the immune function of mice, and the tolerance level of toxicities, which is quite different from human beings [30].

Perhaps the construction of multi-system models for tumor organoids is one of the possible solutions. Skarda et al. [31] constructed an organoid system on an organ-on-a-chip platform, containing heart, lung, and liver organoids. The system facilitates the evaluation of pharmacological effects and toxic responses of drugs on the whole organ. This invention provides a realistic basis for our above assumptions. To some extent, a systematic organs chip can be understood as an upgraded version of Microfluidic 3D culture. The key to a systematic organs chip lies in reconstructing the complexity of the internal organ system by regulating the arrangement and proportion of multiple cells at the microscale. Organ chips are based on microfluidic technology to precisely regulate the metabolic processes and molecular signal transduction of cells by controlling various factors such as flow rate, concentration, and composition of the culture medium. Combined with biomimetic design, organ chips can provide a physiological environment closer to the body [26].

### Constructing vascularised co-culture models

Nowak-Sliwinska et al. [32] discovered that targeting VEGF improved immune infiltration, implying a correlation between tumour angiogenesis and immune infiltration .Hence, the vascularization of tumors also affects the infiltration effect of CAR-T cells. Nevertheless, the current co-culture model of tumor organoids employed for CAR-T research lacks vascularised structures. Whilst this approach does mimic the hypoxic tumour environment to some extent, it limits the size of the tumor organoids and prevents the process of CAR-T cells emerging from the blood vessel to the tumour site from being realistically demonstrated. The development of vascularised tumor organoids could offer researchers a new perspective to elucidate the poor infiltration of CAR-T cells in solid tumours.

Vascularized organoid models can be generated through diverse methodologies. In an initial strategy, vascularized organs were implanted into the renal capsular of immunodeficient mice, thereby enabling vascularization through the host's vascular system. This technique was effectively employed by Waston et al. [33]. They successfully construct vascularize small intestine organoids derived from human embryonic stem cells (hESCs) or human induced pluripotent stem cells (hiPSCs). The utilization of co-culture strategies has been demonstrated as a viable approach for the development of organoid vascularisation models, as exemplified in the research conducted by Sun et al. [34]. In their study, hESCs were directed towards the formation of vascular organoids and brain organoids through differentiation into vascular precursor cells and neuronal precursor cells, which were subsequently co-cultured. This co-culture process facilitated the accurate replication of the vascular architecture within the brain. The construction strategy of Cakir et al. [35] is more interesting. Initially, they observed a strong correlation between the human ETS variant 2 (ETV2) transcription factor and the development of human vascular endothelial cells. Expanding upon this observation, they crafted hESCs that expressed the ETV2 transcription factor and successfully induced it into a human cortical brain organoid with vascular pedicle. Finally, researchers have successfully created vascularised brain organs-on-chips through the utilization of microfluidics [36]. Microfluidics allows for more accurate construction of physiologically similar 3D microenvironments and the creation of a perfusable vascular network for brain organoid vascularisation. This advancement facilitates the administration of drugs or modified immune cells into the organoids.

### Expand the variety of tumor organoids and standardisation of culture procedures

Over the years, there has been significant development in the field of tumor organoids, and various types of patient-derived organoids(PDOs) have been developed (including colorectal cancer [1], breast cancer [37], hepatocellular carcinoma [38], and non-small cell lung cancer [39]). The origins of PDOs have become more diverse, as exemplified by Gao et al. [40] successful generation of prostate cancer organoids from circulating tumor cells in patients, as well as the establishment of PDOs from bronchoalveolar lavage fluid [41, 42]. However, there is still a dearth of organoid models for the rare malignancies typified by neuroendocrine tumours and tumours with non-epithelial origins.

**Fig. 2:**
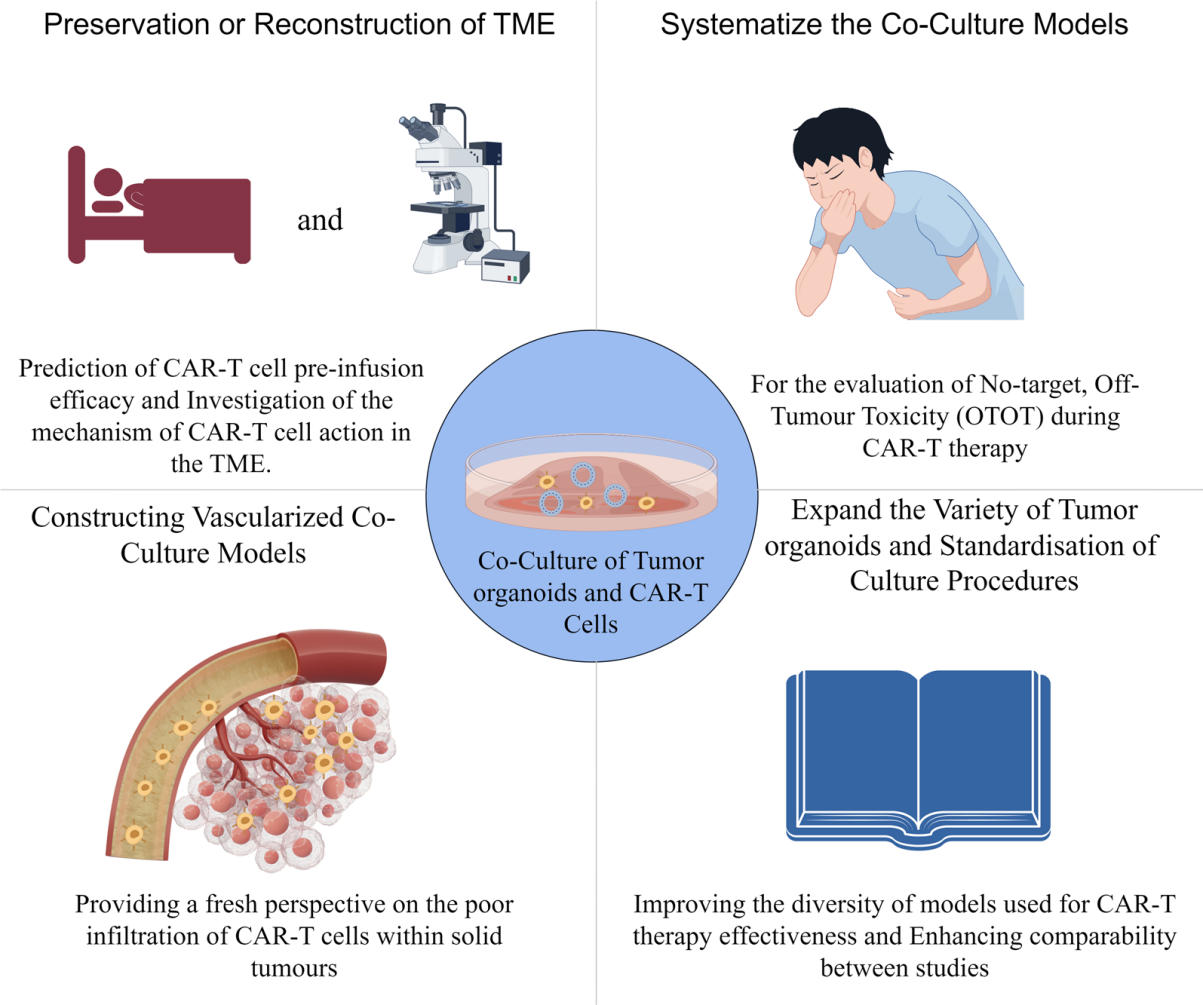
Potential application value of modified tumor organoids and CAR-T cell co-culture models (by Figdraw.)

Standardised culture procedures are of paramount importance. Unstandardised sampling procedures can reduce tumour tissue activity and thus affect the success rate of tumor organoids culture. Cultivating tumour specimens adulterated with normal epithelial tissues can lead to slow growth of tumor organoids. Additionally, the incorrect addition of growth factor/pathway inhibitors may cause induced clonal selection of tumouroid organs. Tumor organoids achieve a high level of preservation of genetic material and specific markers of the primary tumour tissue, so how can tumor organoids ensure that these characteristics are not altered during the cell culture process? At present, the culture process of tumor organoids still needs further standardization (Fig. 2).

### Conclusions and prospects

In summary, we introduce a novel tumor model that can be used for solid tumor CAR-T therapy research, which addresses some of the limitations of commonly used preclinical models in CAR-T research. Currently, some researchers have used it for the validation of anti-tumor effects on new target CAR-T cells, optimized CAR-T cells, and exploration of new combination therapy strategies. The limitations of the current co-culture models were identified and proposed optimization suggestions from five aspects, including reconstructing or preserving TME, systematization, standardization and expanding the tumor organoids resource library, in combination with new advances in organoid technologies.

Improvements to the co-culture model could broaden its applicability to various research scenarios, thus making it a formidable auxiliary tool for personalised precision medicine and basic CAR-T therapy research. High-throughput screening of clinical drugs utilizing tumour organoids is a conventional application of organoids [43]. However, there has been a gap in the assessment of patient efficacy prior to CAR-T cell infusion during oncological treatment. The PDOs achieve a high level of preservation for primary tumour tissue characteristics. Various techniques enable the reconstruction or preservation of TME. This model is expected to be an effective addition to precision treatment, including CAR-T therapy. Wang et al. [44] developed microorganoids spheroids (MOSs) using emulsion microfluidics. Additionally, an automated MOS seeding, processing and imaging system has also been developed. Based on this efficient therapeutic analysis platform, I believe that the aforementioned prospects will soon be achievable. Due to the presence of TME, which leads to a poor response of CAR-T therapy to solid tumours, our knowledge of the mechanisms by which TME affects CAR-T cells is currently limited. However, tumor organoids co-culture models with preserved TME offer a realistic platform for further in-depth research in this area. Inadequate local infiltration of CAR-T cells following infusion is a frequent issue encountered by patients undergoing CAR-T cell therapy. It is closely related to the presence of TME, but there is also a correlation between the physiological process of CAR-T cells drilling out of blood vessels. The phenomenon of vascular mimicry has been confirmed in various malignant tumors [45], so what impact will this phenomenon have on the infiltration of CAR-T cells? This presents an interesting area for exploration. The construction of a co-culture model for vascularization provides strong support for the study of the interaction between CAR-T cells and tumor blood vessels.

Finally, the utilisation of tumor organoids co-culture models in scientific research in oncology treatments is not limited to CAR-T therapies. Schnalzger et al. [14] utilised colorectal cancer organoids to carry out killing toxicity assessments of CAR-NK. Recently, CD19-CAR-T cells have been used for the treatment of systemic lupus erythematosus, achieving encouraging therapeutic effects [46, 47]. At the same time, some research teams have successfully used organ chip technology to construct and simulate organoid models of autoimmune diseases [48, 49]. The good news from these two aspects has given us great inspiration—let's imagine that with the development of autoimmune disease organoid models, the autoimmune disease organoids and CAR-T cell co-culture models will become a preclinical model with great potential, just like the tumor organoids and CAR-T cell co culture models, widely used in preclinical research, pre infusion efficacy evaluation, and other aspects, which will benefit a large patient population. In brief, co-culture models are expected to see extensive use in future scientific research on adoptive cell therapy (ACT). The potential for growth in co-culture models remains high, and researchers are encouraged to conduct thorough explorations of such opportunities.

## Data Availability

Not applicable.
